# Nematode CDC-37 and DNJ-13 form complexes and can interact with HSP-90

**DOI:** 10.1038/s41598-021-00885-4

**Published:** 2021-11-01

**Authors:** Lukas Schmauder, Eva Absmeier, Alexander Bepperling, Katalin Barkovits, Katrin Marcus, Klaus Richter

**Affiliations:** 1 Department of Chemistry, Center for Integrated Protein Research, Technische Universität München, Lichtenbergstr. 4, 85748 Garching, Germany; 2 Medizinisches Proteom-Center, Ruhr-Universität Bochum, Gesundheitscampus 4, 44801 Bochum, Germany; 3 Medical Proteome Analysis, Center for Protein Diagnostics (PRODI), Ruhr-University Bochum, Gesundheitscampus 4, 44801 Bochum, Germany

**Keywords:** Protein folding, Chaperones, Mass spectrometry, Electrophoresis

## Abstract

The molecular chaperones Hsc70 and Hsp90 are required for proteostasis control and specific folding of client proteins in eukaryotic and prokaryotic organisms. Especially in eukaryotes these ATP-driven molecular chaperones are interacting with cofactors that specify the client spectrum and coordinate the ATPase cycles. Here we find that a Hsc70-cofactor of the Hsp40 family from nematodes, DNJ-13, directly interacts with the kinase-specific Hsp90-cofactor CDC-37. The interaction is specific for DNJ-13, while DNJ-12 another DnaJ-like protein of *C. elegans*, does not bind to CDC-37 in a similar manner. Analytical ultracentrifugation is employed to show that one CDC-37 molecule binds to a dimeric DNJ-13 protein with low micromolar affinity. We perform cross-linking studies with mass spectrometry to identify the interaction site and obtain specific cross-links connecting the N-terminal J-domain of DNJ-13 with the N-terminal domain of CDC-37. Further AUC experiments reveal that both, the N-terminal part of CDC-37 and the C-terminal domain of CDC-37, are required for efficient interaction. Furthermore, the presence of DNJ-13 strengthens the complex formation between CDC-37 and HSP-90 and modulates the nucleotide-dependent effects. These findings on the interaction between Hsp40 proteins and Hsp90-cofactors provide evidence for a more intricate interaction between the two chaperone systems during client processing.

## Introduction

Cdc37 is a well-known co-chaperone of Hsp90, which is required for the stable folding of many client kinases during their maturation. It consists of 3 structural domains, namely the N-terminal, central and C-terminal domain^1^. Crystal structures of Hsp90·Cdc37 and biochemical studies on the nematode HSP-90·CDC-37·kinase complexes reveal that the C-terminal domain of phosphorylated Cdc37 initiates the binding to a kinase-substrate, before the N-terminus of Cdc37 and the C-terminus of Hsp90 interact which each other^2–4^. During this process, the ATPase-activity of Hsp90 is inhibited by Cdc37, which blocks Hsp90’s N-terminal ATP binding site thereby holding Hsp90 in an open conformation and assisting the loading of kinase clients onto the chaperone^5,6^. Binding of Aha1, another co-chaperone of Hsp90, leads to the dissociation of Cdc37 from the complex and Hsp90 is able to change into the closed ATPase active conformation, eventually leading to a mature kinase^3,7–9^. The detailed mechanism of kinase loading onto the Cdc37·Hsp90 complex still remains unknown.

Instead of forming a stable complex with Hsp90 and Cdc37, kinases can also associate with Hsc70 and Hsp40, leading to degradation by the proteasome if Cdc37 is depleted^10^. Here Hsp40 or in general J domain-containing proteins stimulate the hydrolysis activity of Hsc70 by binding to its ATP-bound state. Various nucleotide exchange factors like BAG domain-containing proteins then trigger nucleotide release by competing with the highly diverse J domain-proteins, leading to a strong ATP turnover if both are present^11^.

In *Caenorhabditis elegans* (*C. elegans*), there are three cytosolic Hsp40-like J domain proteins, namely DNJ-12, DNJ-13 and DNJ-19 as well as the two Hsp40-like J-proteins DNJ-7 and DNJ-16 from mitochondria and ER, respectively. DNJ-12 and DNJ-19 share full domain conservation with *Escherichia coli* (*E. coli*) DnaJ, which is the namesake of the J-protein family.

DNJ-13 shares its N-terminal J-domain and its Gly/Phe-rich domain with DnaK, but the Cys-rich domain is replaced by a Met/Gly-rich domain^12^. The Gly/Phe-rich domain seems particularly interesting due to its involvement in the ATP-dependent substrate binding of HSP-70^13,14^. A study by Perales-Calvo et al. found that the Gly/Phe-rich domain of DnaJ plays a crucial role in the conformational recognition of substrate proteins^15^, and in the ability of DnaJ to interact with folded substrates. Further, the Cys-rich domain of DnaJ binds denatured substrates and stabilizes the DnaK-substrate complex^16–18^. It is assumed that this domain also controls the J-protein interaction with other proteins, and thereby supports the chaperone functions^18–21^.

The C-terminus contains two beta-barrel-topology domains CTD I and CTD II. In addition, a separate C-terminal dimerization-domain also influences the affinity for client proteins^22,23^. Especially CTD I is thought to play an important role in client binding, due to a hydrophobic pocket^21,24^ and the outbound Gly/Met-rich domain^16–18^. Based on the importance of client binding in vivo, the deletion of the C-terminus of DnaJ-like proteins lead to non-viable mutants^25^. For the CTDII of J-proteins no function is known yet, but it precedes the C-terminal dimerization domain.

Both chaperone systems, Hsp70 and Hsp90, are involved in proteostasis control and specific folding of client proteins. Their cofactors may frequently interact during these functions within the large protein assemblies. To reveal potential interactions between cofactors, we employed protein cross-linking and analytical ultracentrifugation on the Hsp90-cofactor CDC-37 in order to investigate possible interaction partners and further analyze the crosslinked proteins by mass spectrometry.

## Material and methods

### Cloning, protein expression and purification

The pET28b plasmid was used as expression vector with the cDNA of either DNJ-13, CDC-37 and the respective fragments of CDC-37 (ΔN & ΔC), subcloned after the N-terminal His_6_ tag. For expression, transformed *E. coli* BL21-CodonPlus(DE3)RIL cells were grown to an OD_600_ of 0.6 at 37 °C. Protein production was induced by adding 1 mM isopropyl 1-thio-β-D-galactopyranoside and further incubation at 20 °C. Cells were harvested and subsequently disrupted in a TS 0.75 cell disruption instrument (Constant Systems Ltd., Northants, UK). The His_6_-tagged proteins were trapped on a HisTrap FF 5-mL affinity column (GE Healthcare) in 40 mM HEPES/KOH, pH7.5, 20 mM KCl, 1 mM DTT and eluted with buffer containing 300 mM imidazole. ResourceS ion-exchange chromatography and size exclusion chromatography on a 16/60 Superdex 75 HiLoad column (GE Healthcare) were subsequently performed. Proteins were stored and measured in a buffer containing 40 mM HEPES/KOH, pH7.5, 20 mM KCl, 1 mM DTT. The quality of each purified protein was confirmed by SDS-PAGE and mass spectroscopy on a Bruker UltraFlex III MALDI-TOF instrument (Bruker, Massachusetts, USA).

### Fluorescence labeling of CDC-37

Labeled CDC-37 (*CDC-37) was generated by fluorescently labeling 0.5 mg CDC-37 at its cysteine residues with a twofold molar excess of ATTO 488 C_5_-maleimide (ATTO-TEC, Germany) for 2 h at room temperature in a buffer of 40 mM HEPES/KOH, pH7.5 and 20 mM KCl. The reaction was stopped by the addition of 20 mM DTT and *CDC-37 was subsequently dialyzed against the same buffer to remove free label. The degree of labeling as well as the concentration of the protein was determined by UV/VIS spectroscopy using the following equations:

$$ {\text{A}}_{{{\text{Protein}}}} = {\text{ A}}_{{{28}0}} - {\text{A}}_{{{5}00}} \cdot \, \left( {{\text{CF}}_{{{28}0}} } \right) $$

$$ {\text{DOL }} = {\text{A}}_{{{5}00}} \cdot {\text{MW}}/\left[ {{\text{protein}}} \right] \cdot \varepsilon_{{{\text{dye}}}} $$

where CF_280_ = 0.09 and ɛ_dye_ = 90,000 M^−1^ cm^−1^ according to the manufacturer.

### Analytical ultracentrifugation

Analytical ultracentrifugation (AUC) experiments were carried out in a Beckman ProteomeLab XL-A analytical ultracentrifuge (Beckman Coulter, Brea) equipped with an AVIV AU-FDS fluorescence detection system (Aviv Biomedical, Lakewood, NY) and a Ti-50 rotor (Beckman Coulter, Brea) as described previously^26^. For binding analyses, 300 nM of *CDC-37 were sedimented at 42,000 rpm and 20 °C in the absence or presence of 2 µM binding partners. Sedimentation experiments were performed in 40 mM HEPES/KOH pH 7.5, 20 mM KCl, 1 mM DTT and 5 mM MgCl_2_. dc/dt distributions were visualized with the program Sedview^27^ and the customized in-house script diffUZ was used for flexible scan range selection, for normalization of the data and for generation of the plots as described previously^11,28^. Fits to Gaussian functions were made in OriginPro (Version 2018b) (OriginLab Corporation, Northampton, MA, USA; https://www.originlab.com/origin). Each assay was measured in triplicates and a representative graph is plotted.

Sedimentation experiments of non-labeled proteins were performed in a similar manner using a concentration of 0.5 mg/ml protein and an UV/Vis optical system (Beckman Coulter, Brea). dc/dt plots were generated with the in-house script diffUZ as described in^29^. 2DSA analysis in UltraScan III Version 4.0 (https://ultrascan3.aucsolutions.com/)^30^ was employed to get information on the heterogeneity of the sample and to derive sedimentation coefficients, diffusion coefficients and molecular weights for the species.

### K_D_ determination

Fluorescence AUC was deployed to determine the approximate K_D_ of the DNJ-13·CDC-37 complex, using 100 nM *CDC-37 with increasing DNJ-13 concentrations (0–10 µM). Saturated complexes are represented by peaks with the highest sedimentation coefficients, which were plotted against the corresponding DNJ-13 concentrations. The K_D_ was then derived by fitting a Michaelis–Menten function in OriginPro (Version 2018b) (OriginLabs, Northampton, USA) onto the data points.

### Crosslinking of DNJ-13·CDC-37 complexes

Crosslink experiments were performed using deuterium isotope labeled BS3-H_12_/D_12_ and DSSG- H_6_/D_6_ (Creative Molecules Inc.) as crosslinking reagents. 16 µM DNJ-13 and 16 µM CDC-37 were mixed in a total volume of 50 µL (40 mM HEPES/KOH, pH7.5, 20 mM KCl) and crosslinked with a 32.5-fold molar excess of crosslinker. The reaction was stopped after 20 min at room temperature by the addition of 1 M Tris/HCl pH 8.0. As control, both proteins were also crosslinked alone. SDS-PAGE was used to analyze the crosslinked samples on pre-casted SERVAGel Neutral Gradient pH7.4 (SERVA, Heidelberg, Germany) according to the manufacturer’s instruction. Images have not been cropped to remove lanes, as shown in Supplemental Fig. 1–3. Bands of interest were then cut out and sent to the Medical Proteome Analysis Center for Protein Diagnostics (PRODI, Ruhr-University Bochum, Bochum).

**Figure 1:**
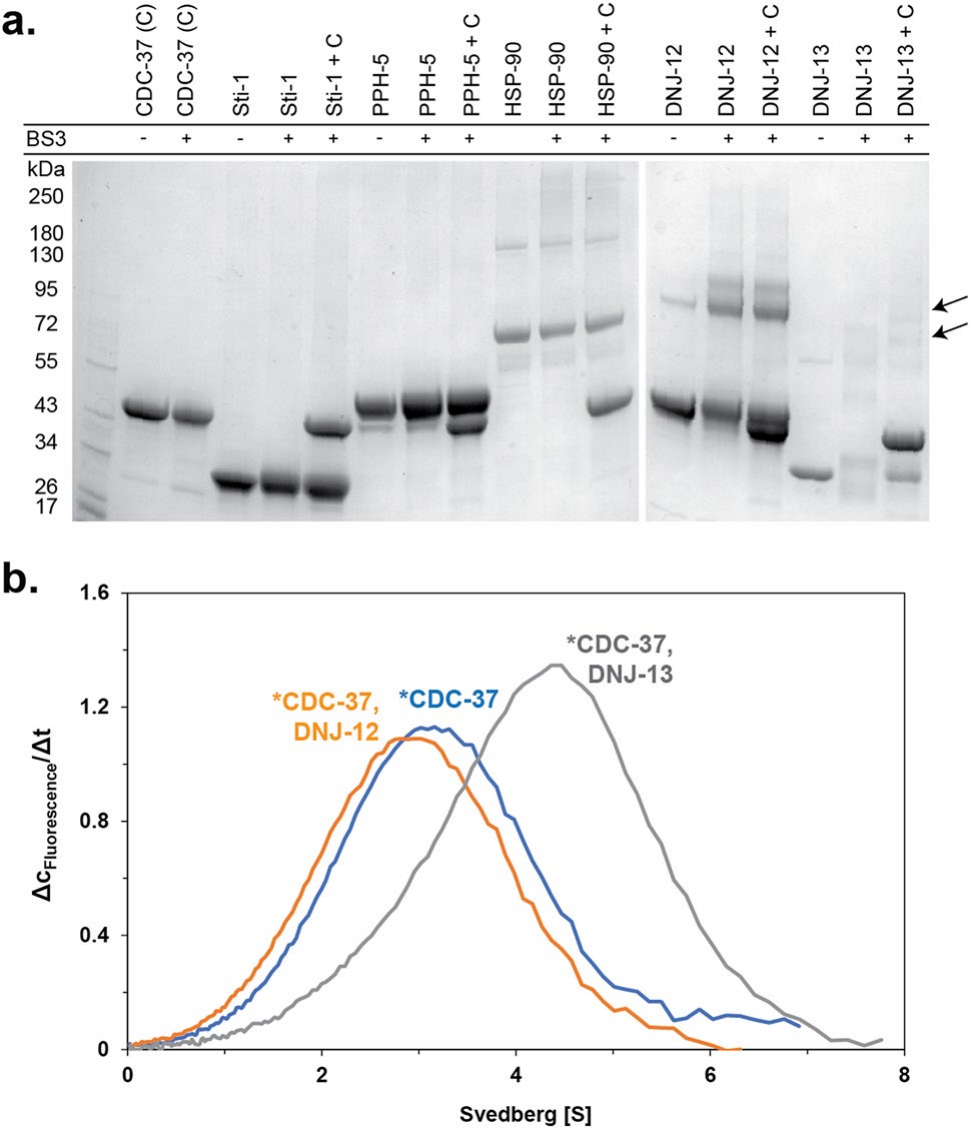
Systematic cofactor CDC-37 interactions. **(a)** Interaction of CDC-37 (C) with and without the crosslinking reagent BS3, as indicated together with the cofactors Sti1, PPH-5, HSP-90, DNJ-12 and DNJ-13. The upper arrowhead indicates the observed crosslinked CDC-37_monomer_·DNJ-13_dimer_ complex, whereas the lower arrowhead indicates a CDC-37_monomer_·DNJ-13_monomer_ complex. (**b)** Sedimentation analysis of labeled CDC-37 (*CDC-37) together with the J domain-containing proteins DNJ-12 and DNJ-13. OriginPro (Version 2018b) was utilized for generating the graph.

### Mass spectrometry

Gel pieces containing crosslinked protein bands were washed and digested in-gel as described previously^31,32^. The peptide concentration was then determined by amino acid analysis (AAA) as described by Plum et al.^33^.

Tryptic peptides were measured by nanoLC-ESI–MS/MS, also as described previously^32,34^. In short, an UltiMate 3000 RSLC nanoLC system (Thermo Scientific, Bremen, Germany) was used with the following solvent system: (A) 0.1% FA; (B) 84% ACN, 0.1% FA. For sample concentration and washing, loading on a trap column (Thermo, 100 μm × 2 cm, particle size 5 μm, pore size 100 Å, C18) at a flow rate of 30 μl/min with 0.1% TFA was performed. Afterwards, the trap column was serially connected to an analytical C18 column (Thermo, 75 μm × 50 cm, particle size 2 μm, pore size 100 Å), and the peptides were separated with a flow rate of 400 nl/min using a solvent gradient of 4% to 40% B for 95 min at 60 °C. 1 h of column washing was performed for equilibration after each sample measurement. The UltiMate 3000 RSLC nanoLC system was on-line connected to the nano-electrospray ionization source of a Q-Exactive HF (Thermo Scientific, Bremen, Germany).

Full MS spectra were scanned in the range 350–1400 m/z with a resolution of 60,000 at 200 *m/z* (AGC target 3e6, 80 ms maximum injection time). Capillary temperature was set to 275 °C and spray voltage to 1500 V (positive mode). Lock mass polydimethylcyclosiloxane (m/z 445.120) was used for internal recalibration. The m/z values initiating MS/MS were set on a dynamic exclusion list for 30 s and the 10 most intensive ions (charge 2 + to 6 +) were selected for fragmentation. MS/MS fragments were generated by higher-energy-collision-induced dissociation and the fragmentation was performed with 28% normalized collision energy. The fragment analysis was performed in an orbitrap analyzer with resolution 30,000 at 200 *m/z* (AGC 1e6, maximum injection time 120 ms).

### Identification of crosslink sites

To confirm the presence of all proteins, the raw data was analyzed with MaxQuant 1.5 (https://maxquant.org/maxquant/)^35^ and tables for the crosslinked proteins were obtained from all unmodified peptides and the complete peak lists. The peak lists also yielded intensity values and elution times for each peptide and were then imported into the in-house script xMASS^29^ and analyzed as previously described^31^.

A similar analysis on the same raw data was performed in pLink2.0 (http://pfind.ict.ac.cn)^36^ with the following settings: Peptide mass range: 600–6000 Da; Peptide length: 6–60 AA; precursor tolerance: ± 15 ppm; fragment tolerance: ± 15 ppm; filter tolerance: ± 15 ppm; FDR < 5% at PSM level. Reported crosslinked spectra were filtered to a mass error < 2 ppm and then compared to the xMASS outputs. The most prominent confirmed hits are listed in Table 1. Sequence alignments of template and target proteins were performed in CLUSTALW (http://www.clustal.org/clustal2/)^37^.

**Table 1:** Most prominent unique hits in the crosslinked sample. Grey colored parts are obtained from DSSG crosslinked samples while white parts are obtained from BS3 crosslinked samples. **a)** Intermolecular crosslink pairs of sample #4 (Complex at approx. 135 kDa). **b)** Intermolecular crosslink pairs of sample #5 (Complex > 245 kDa). **c)** Both Inter- and intramolecular crosslinked pairs in the reference sample #3 (DNJ-13 dimer).

Peptide 1	Peptide 2	Protein 1	Protein 2
**(a)**			
EAGAENKFK	GTTSKK	DNJ-13 (44)	CDC-37 (60)
MEQEKIDK	YHPDKNK	CDC-37 (50)	DNJ-13 (35)
GKDYYKVLGISK	EKGTTSK	DNJ-13 (3)	CDC37 (55)
KMKITR	KPQAPK	DNJ-13 (179)	CDC-37 (132)
MEQEKIDK	YHPDKNK	CDC-37 (50)	DNJ-13 (35)
AYRKMALK	MEELEKK	DNJ-13 (26)	CDC-37 (67)
MGKDYYK	GTTSKK	DNJ-13 (1)	CDC-37 (60)
MPIDYSK	GTTKKMK	CDC-37 (1)	DNJ-13 (176)
AYRKMALK	GTTSKK	DNJ-13 (26)	CDC-37 (60)
GLPNPKSPSHR	PIDYSK	DNJ-13 (299)	CDC-37 (2)
KEAELEEK	MGKDYYK	CDC-37 (97)	DNJ-13 (3)
LERMAEKK	DKPHPK	CDC-37 (44)	DNJ-13 (235)
**(b)**			
KFEAAEPVYMK	MGKDYYK	CDC-37 (241)	DNJ-13 (1)
MEQEKIDK	YHPDKNK	CDC-37 (50)	DNJ-13 (35)
MEELEKKLAAADVTDK	KMALKYHPDK	CDC-37 (67)	DNJ-13 (30)
MEQEKIDK	YHPDKNK	CDC-37 (50)	DNJ-13 (35)
**(c)**			
DYYKVLGISK	GATDDEIKK	DNJ-13 (7)	DNJ-13 (21)
GATDDEIKK	YHPDKNK	DNJ-13 (21)	DNJ-13 (35)
GLPNPKSPSHR	GATDDEIKK	DNJ-13 (299)	DNJ-13 (21)
KVMTDNAQR	EAGAENKFK	DNJ-13 (183)	DNJ-13 (44)
DYYKVLGISK	EAGAENKFK	DNJ-13 (7)	DNJ-13 (44)
NKEAGAENK	GKDYYK	DNJ-13 (37)	DNJ-13 (3)
KIYDQFGEEGLK	GATDDEIKK	DNJ-13 (62)	DNJ-13 (21)
VLGISKGATDDEIKK	EAGAENKFK	DNJ-13 (13)	DNJ-13 (44)
EIAEAYDVLSDDKKK	KIYDQFGEEGLK	DNJ-13 (59)	DNJ-13 (62)
VEKISLK	EGSDIKR	DNJ-13 (252)	DNJ-13 (248)
EAGAENKFK	GATDDEIKK	DNJ-13 (44)	DNJ-13 (21)
DYYKVLGISK	EGSDIKR	DNJ-13 (7)	DNJ-13 (248)

### Molecular modelling and docking calculations

DnaJ of *Thermus thermophilus* (PDB 4J80)^38^ served as homology template for DNJ-13 using the Chimera 1.13.1 interface to MODELLER (https://www.cgl.ucsf.edu/chimera/)^39,40^. Likewise for the generation of a CDC-37 homology model, human Cdc37 (PDB 5FWL)^41^ was used. Docking of both homology models was performed in HADDOCK 2.4 (https://wenmr.science.uu.nl/haddock2.4/)^42^. Lysine residues, which were identified by crosslinking, were defined and a distance restraint of 30 Å was enforced on those residues in expert mode. PyMOL 2.5 (https://pymol.org/2/) was used to generate space-filling and ribbon models and crosslinks were visualized with the PyMOL plugin PyXlinkViewer (https://github.com/BobSchiffrin/PyXlinkViewer)^43^.

## Results

### General interaction between CDC-37 and DNJ-13

Several interactions between Hsp90-cofactors had been observed in the yeast model system^44^. We aimed at identifying similar interactions of nematode CDC-37 and therefore investigated, whether the Hsp90-cofactor CDC-37 can potentially interact with other cofactors. We first performed a crosslink screening of several Hsc70 cofactors utilizing BS3 as a crosslinking reagent (Fig. 1a). A potential interaction between CDC-37 and the J domain-containing protein DNJ-13 (bands marked by the two arrowheads in Fig. 1a) was revealed, which to our knowledge was unknown until now. The upper band equals a molecular mass, comparable to one CDC-37 monomer and a DNJ-13 dimer, whereas the lower band compares to a CDC-37 monomer together with one DNJ-13 monomer. To confirm these findings, we then employed sedimentation velocity analytical ultracentrifugation (SV-AUC) of both proteins (Fig. 1b).

Fluorescently labeled CDC-37 had been generated before to investigate the interaction of CDC-37 with other cofactors of Hsp90 in the absence and presence of Hsp90 itself. During these studies, large parts of the Hsp90-cycle with Hsp90 could be reconstituted under experimental conditions^4,45^. We also investigated whether the addition of Hsp40 proteins influences the hydrodynamic properties of the fluorescently labeled *CDC-37. Indeed, DNJ-13 leads to a strong shift in sedimentation coefficient from 2.8 S to 5.3 S, implying an interaction between CDC-37 and DNJ-13 and the formation of a protein complex between these two proteins (Fig. 1b). We aimed at understanding, whether this complex is specific to DNJ-13, or whether other Hsp40 proteins likewise form these assemblies. Addition of DNJ-12 instead did not lead to a likewise increase in size and therefore the formation of this complex appears to be specific for DNJ-13 as suggested by the crosslinking experiment.

### Stoichiometry of one DNJ-13 dimer in complex with one CDC-37

**Figure 2:**
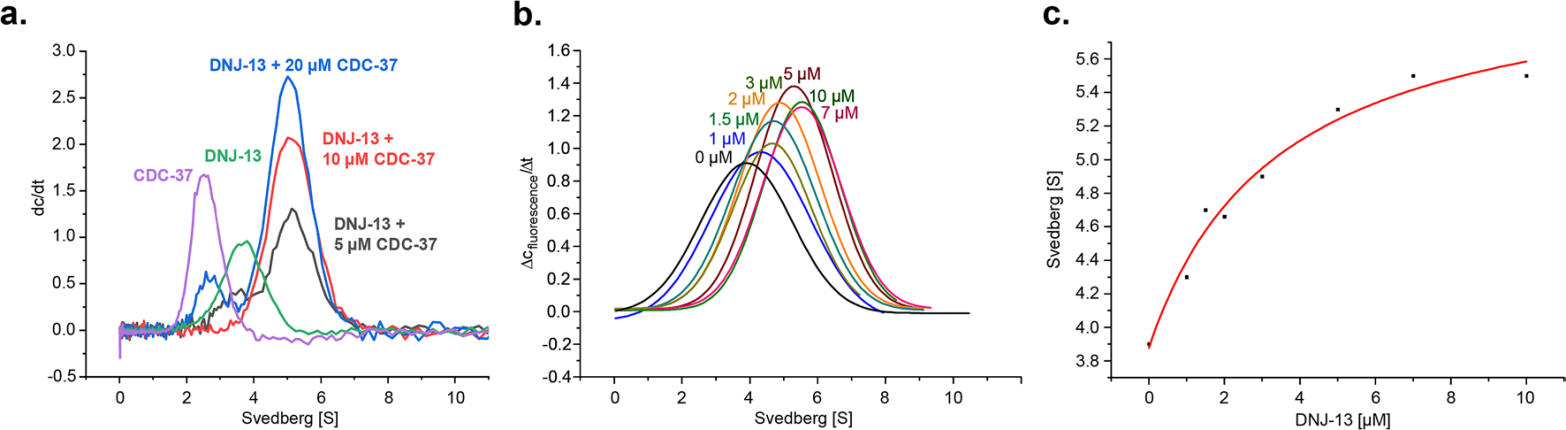
KD and stoichiometry determination. **(a)** Absorbance sedimentation velocity analysis of CDC-37 titration to 10 µM DNJ-13 in substoichiometric, equal and excess concentrations, as indicated. CDC-37 (purple) and DNJ-13 (green) serve both as control, as well as assistance for the indication of unbound protein used in the titration. (**b)** Titration of varying DNJ-13 concentrations in AUC experiments to 100 nM *CDC-37, as indicated. (**c)** Kinetics of CDC-37/DNJ-13 binding based on titrated DNJ-13 concentrations and measured sedimentation coefficient. Graphs were generated in OriginPro (Version 2018b).

While AUC with fluorescently labeled proteins is a powerful tool to analyze the oligomerization state as well as the association of labeled proteins with putative binding partners by determination of the hydrodynamic properties, complex formation best is confirmed with unmodified proteins. To ensure that the interaction between CDC-37 and DNJ-13 is not due to the attached label, an AUC experiment was performed with non-labeled CDC-37 and three different concentrations of DNJ-13 (Fig. 2a). The complex was formed with the same sedimentation coefficient as in the fluorescence SV-AUC measurement (Fig. 1b), confirming that the interaction is not caused by the labeling of CDC-37 with the fluorophore. The obtained sedimentation coefficients for CDC-37 alone and CDC-37 in complex with DNJ-13 were approximately the same as in the fluorescence experiments.

To determine the stoichiometry of the complex, absorbance AUC was employed. Three different conditions were compared: one with a substoichiometric concentration of CDC-37, one with an equal concentration of CDC-37 and DNJ-13 dimer, and one with a twofold excess of CDC-37 (Fig. 2a). In the first case, all CDC-37 was bound in complex with the DNJ-13 dimer and a peak with the sedimentation coefficient of the DNJ-13 dimer was still visible (Fig. 2a, black trace). The obtained mass for the second peak based on UltraScan 2DSA-analysis hinted at a complex of one molecule CDC-37 with a DNJ-13 dimer. The second condition showed almost solely a peak for the DNJ-13 dimer with one CDC-37 in complex. In the third condition a peak corresponding to free CDC-37 and a peak for the CDC-37·DNJ-13-dimer complex was visible (blue trace, Fig. 2a).

### Affinity of the complex between CDC-37 and DNJ-13

We next set out to determine the approximate K_D_ of the protein complex. To this end, we employed AUC with fluorescence detection, since the affinity of the protein complex may be too low for the fairly high protein concentrations needed for UV/Vis detection during AUC. The determination thus was carried out with 100 nM AlexaFluor 488-labeled CDC-37 (*CDC-37) and DNJ-13 protein at concentrations ranging from 0 to 10 µM (Fig. 2b). The K_D_ was then estimated from the concentrations of free CDC-37 and CDC-37 in complex with DNJ-13 (Fig. 2c). Saturation of complex formation could be obtained as no further increase in sedimentation coefficient was observed for the highest DNJ-13 concentrations employed. This also implies that the interaction is happening at a defined site and the complex was found to have an approximate K_D_ of 3 µM, which indicates an intermediate affinity between the two proteins.

### Identification of binding sites with crosslinks and AUC for CDC-37 variants

**Figure 3:**
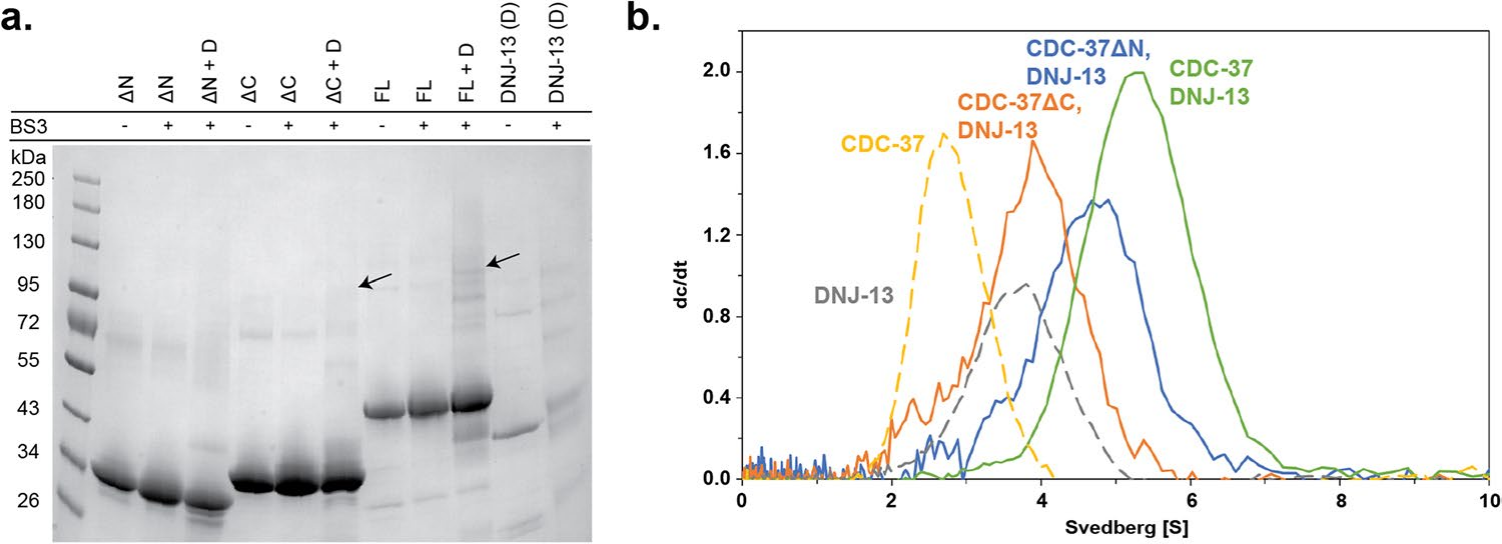
CDC-37 fragment interaction with DNJ-13. **(a)** SDS-PAGE of crosslinked DNJ-13 (D) together with CDC-37ΔN (ΔN), CDC-37ΔC (ΔC) or full-length CDC-37 (FL). Arrowheads indicate the observed complexes. (**b)** Sedimentation analysis of DNJ-13 with either CDC-37ΔN, CDC-37ΔC or full-length CDC-37 as indicated. Shifting sedimentation coefficients are put into perspective by both controls, DNJ-13 (dashed grey) and CDC-37 (dashed yellow) respectively. OriginPro (Version 2018b) was utilized for generating the graph.

Next, we aimed at addressing the binding sites of the two molecules. To confirm the relevance of individual domains for the interaction between the two proteins, deletion mutants of CDC-37 were made deleting either the N-terminus or the C-terminus of the Hsp90-cofactor. To investigate whether the CDC-37 variants are still able to bind full-length DNJ-13 the crosslinking reaction was repeated with CDC-37 and both fragments (Fig. 3a). Several other bands were likewise observed in the isolated proteins and therefore only those bands were considered, that were specific for the setups, in which both proteins were mixed. The mixture, where a C-terminal deleted CDC-37 was used could still perform the cross-linking reaction, implying that the N-terminal part of CDC-37 is capable of performing this reaction. Furthermore, absorbance AUC experiments were performed with the fragments in similarity to the experiments with full-length CDC-37 (Fig. 3b). Both fragments did not interact with the same strength as the full-length CDC-37 protein, implying that both termini may be involved in the binding reaction. However, a stronger increase in the sedimentation coefficient was observed for DNJ-13 + CDC-37ΔN (5.2 S) compared to DNJ-13 + CDC-37ΔC (4.2 S), which implies a stronger interaction as indicated by the titration of CDC-37 with DNJ-13 before. Nevertheless, for both deletion fragments, the peaks are fairly broad, indicating a dynamic binding mode and a weaker interaction. Based on the SV-AUC data most of the binding site seems to be located at the C-terminus of CDC-37, despite the identification of the crosslink at the N-terminus. Nevertheless, it is obvious from the AUC results that both regions contribute and the identification of the crosslink at the N-terminus may represent only one of the two binding sites.

### CDC-37/DNJ-13 in complex with HSP90

**Figure 4:**
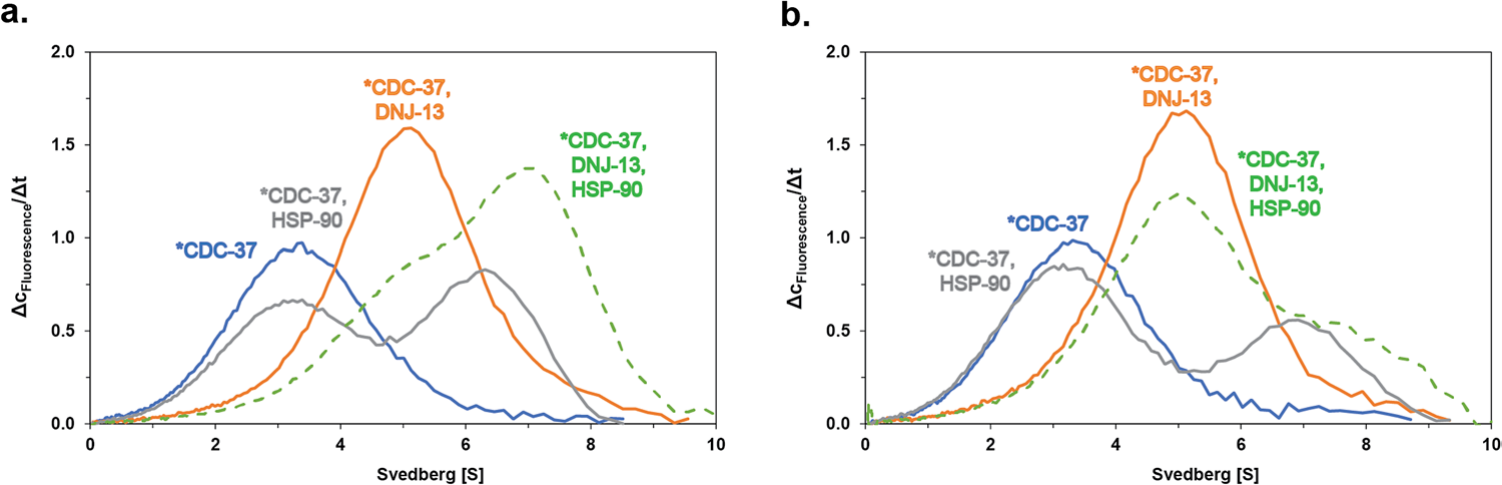
Interaction of HSP-90 with the CDC-37·DNJ-13 complex. **(a)** Sedimentation analysis of the binary complex, consisting of the either *CDC-37 and DNJ-13 (orange), *CDC-37 and HSP-90 (grey) or the ternary CDC-37·DNJ-13·HSP-90 complex (dashed green). (**b)** Effect of ATPγS addition to both binary and ternary complex, similar to **(a)**. Graphs were generated in OriginPro (Version 2018b).

We also aimed to determine, whether the CDC-37/DNJ-13 complex still can bind to HSP-90, for which CDC-37 acts as a co-chaperone during kinase maturation. Employing the fluorescently-labeled CDC-37 protein, AUC was performed to see whether the DNJ-13 complex binds to open or closed forms of HSP-90 (Fig. 4a). To this end, ATPγS was supplemented into the binding reaction to induce the closed conformation of HSP-90 (Fig. 4b). In the presence of ATPγS the equilibrium is shifted towards unbound CDC-37 as described previously. The equilibrium is shifted towards the ternary complex for the open form of HSP-90, but no influence of the nucleotide on the formation of the DNJ-13·CDC-37 complex is observed. The ternary complex between the three proteins can be clearly observed at 7 S, with only little residual CDC-37·DNJ-13 complexes left unbound at the employed concentration of HSP-90. Addition of nucleotides to these complexes releases CDC-37·DNJ-13 complexes from HSP-90, indicating that the regulation of the CDC-37-Hsp90-interaction is still functional, when DNJ-13 is associated with CDC-37. Furthermore, no indication was seen that the presence of DNJ-13 dramatically changes the interaction between the complexed CDC-37 and Hsp-90. CDC-37 binds mainly the open form of HSP-90 but there is still unbound CDC-37 in the nucleotide-closed or the open state. In the presence of DNJ-13, all CDC-37 is bound to either the HSP-90 containing complex or to DNJ-13, indicating that CDC-37 has a stronger affinity to DNJ-13 than towards its chaperone HSP-90.

### Structural organization of the CDC-37·DNJ-13 complex

**Figure 5:**
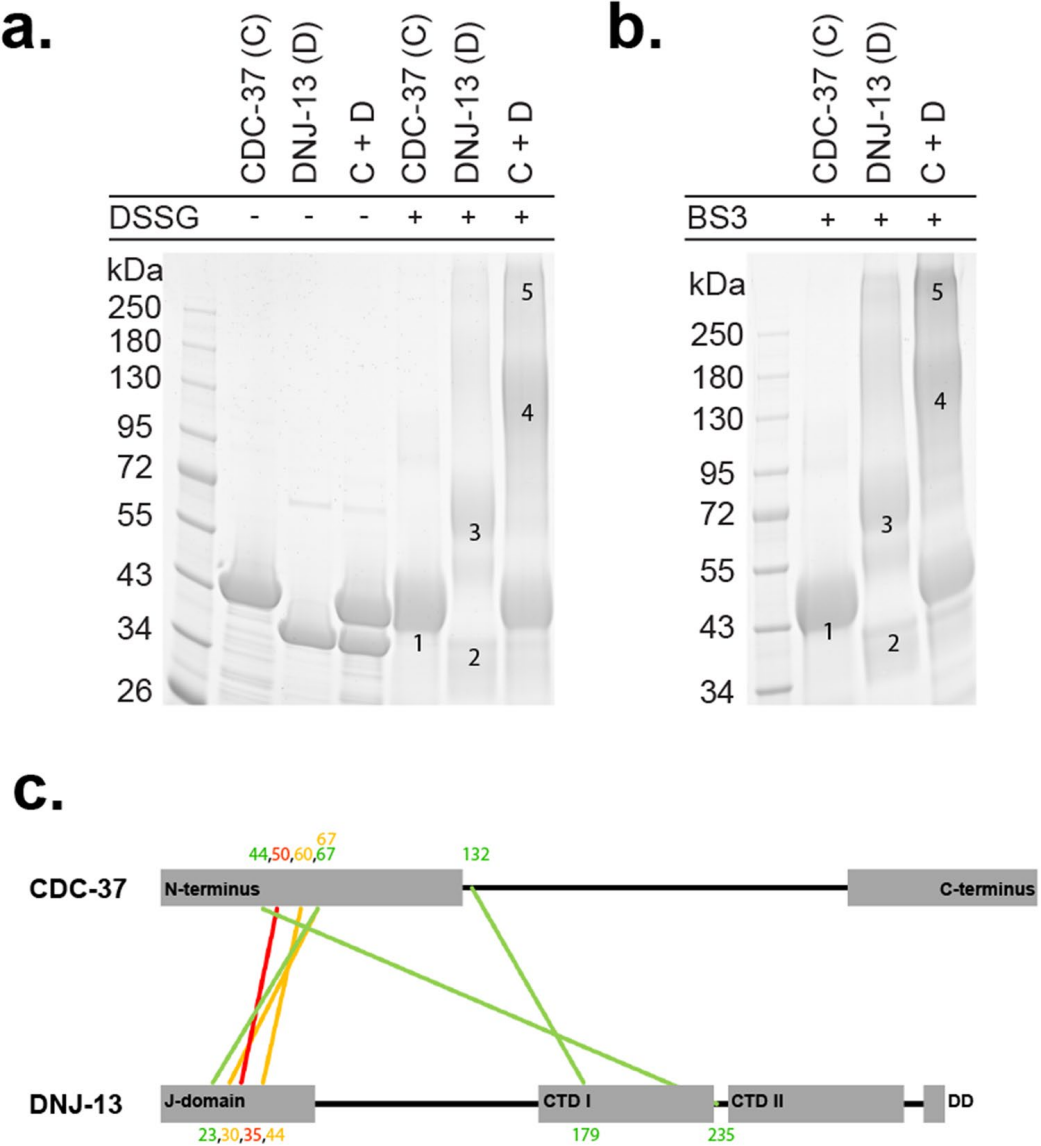
Mass spectrometry crosslink analysis. **(a)** Samples used for MS-analysis and crosslinked with DSSG. 1 = CDC-37 monomer, 2 = DNJ-13 monomer, 3 = DNJ-13 dimer, 4 = CDC37·DNJ13 complex made out of one CDC-37 and two DNJ-13 molecules, 5 = complex consisting of two CDC37·DNJ13 complexes. (**b)** Samples used for MS-analysis, crosslinked with BS3, with the same naming scheme as in **(a)**. (**c)** Visualization of the crosslinked pairs, obtained from MS-analysis (Table 1). Green lines indicate pairs crosslinked with DSSG; Yellow lines indicate pairs crosslinked with BS3; Red lines indicate the pair crosslinked by both DSSG and BS3.

Next, we analyzed the crosslinked binary complex of CDC-37 and DNJ-13 (Fig. 5a,b) by mass spectrometry to find key crosslink pairs in the complex, which would reveal more structural information.

Datasets generated by MS were analyzed by two independent programs, pLink2.0 and the in-house script xMASS, both yielding comparable results. We then obtained a schematic diagram of the relative protein arrangement in the complex (Fig. 5c) based upon the identified intermolecular crosslinks (Table 1). 6 of the 10 intermolecular crosslinks could be obtained using DSSG as crosslinking compound, the 4 other pairs were linked by BS3. A major contact site was found between CDC-37 peptide MEQEKIDK (AA50) and DNJ-13 peptide YHPDKNK (35), occurring in both datasets. Another possible interaction site was identified between the N-terminus of CDC-37 (AA44) and in between the C-terminal domain I (CTDI) and II (CTDII) of DNJ-13 (AA235). The N-terminus of CDC-37 is known to be involved in client kinase binding and while CTDI is thought to play an important role in client binding, no function is known for the CTDII of J-proteins yet^1,21,24^.

**Figure 6:**
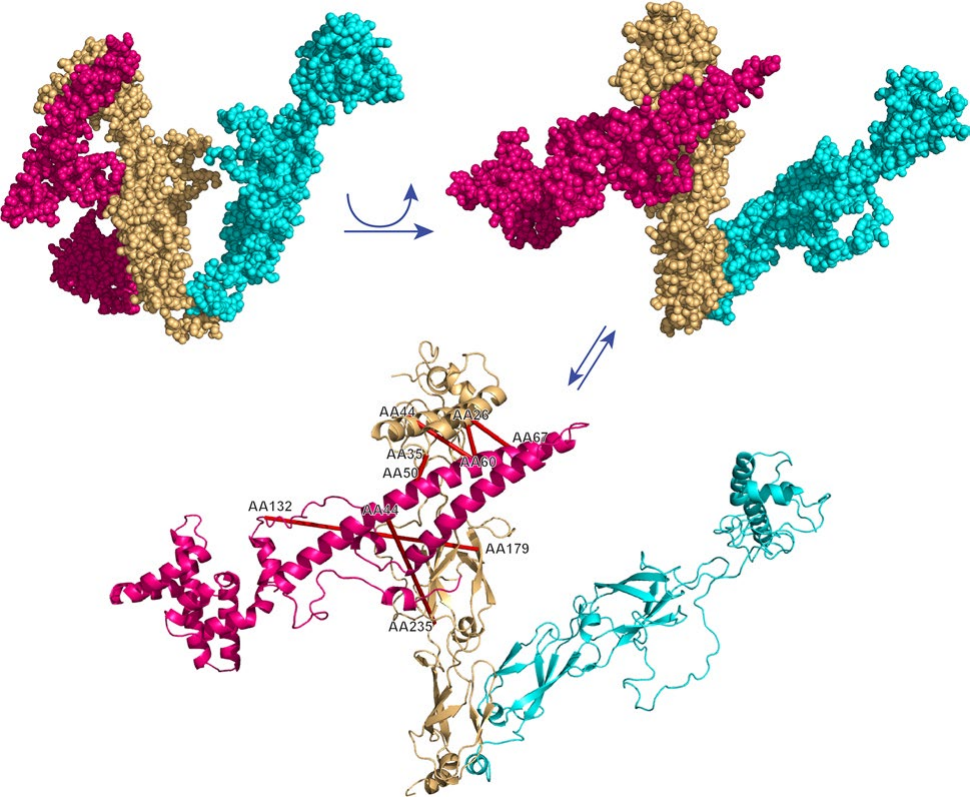
Homology model of the CDC-37·DNJ-13 complex. PDB structure 4J80 served as template for DNJ-13, monomers highlighted in cyan and brown; CDC-37 was modeled after PDB structure 5FWL, highlighted in magenta. Arrows indicate a rotation of the model and/or a swap between visualizations as either space-filling or ribbon model. Crosslinks are depicted as red lines between the ribbon models. Homology models were generated in Chimera 1.13.1, docked with HADDOCK 2.4 and crosslinks were visualized in PyMOL 2.5.

We then calculated a homology model for both nematode CDC-37 and DNJ-13, based on the human homolog Cdc37 (PDB: 5FWL) and bacterial DnaJ (PDB: 4J80) respectively. Finally, we performed an assembly of the proposed CDC-37·DNJ-13 complex, based upon the most prominent intermolecular crosslinks (Fig. 6). Potential differences due to the limited sequence identity of both CDC-37 (37%) and DNJ-13 (31%) to their respective PDB templates, are not respected during homology modeling since most distances between crosslinked positions are in agreement with the 30 Å maximal length of the crosslinker. Crosslink pairs with a distance over 30 Å imply that minor rearrangements of the structure may have to be considered. Since a significant part of Cdc37 is not well resolved in PDB 5FWL further ambiguity exists. Nevertheless, the docking approach indicates that the two cofactors may interact alongside one DNJ-13 dimer with both domains of CDC-37 bound to that subunit.

## Discussion

CDC-37 is known as HSP-90 co-factor during kinase maturation^46,47^. It arrests the ATP-driven molecular clamp mechanism of Hsp90 and thus has an inhibitory effect on the ATPase activity of HSP-90. Even though the partial crystal structure of the Cdc37·Hsp90 complex is solved^5^ and parts of the kinase maturation cycle are understood^48,49^, the question how the kinase is loaded onto the Cdc37·Hsp90 complex still remains unclear. We found that nematode DNJ-13, a well-described Hsc70 cofactor, interacts with the kinase specific Hsp-90 cofactor CDC-37 from nematodes. Altogether our results demonstrate an interaction between the Hsp90 co-chaperone CDC-37 and the J domain-containing protein DNJ-13 and the ability to form a stable complex in vitro. The complex has a K_D_ of approximately 3.4 µM and a stoichiometry of one CDC-37 monomer bound to a DNJ-13 monomer or dimer.

J-proteins, like DNJ-13, are known to form dimers^28^ and CDC-37 interacts as a monomer in the CDC-37/HSP-90 kinase complex^48^. Interestingly, the complex of DNJ-13 and CDC-37 interacts with HSP-90, preferentially with the open state. In these assays, DNJ-13 binds stronger to CDC-37 than to HSP-90. It might be that the client kinase is further modulating the interaction affinities if present. The observed interaction between CDC-37 and DNJ-13 is interesting, as it links the CDC-37/HSP-90/Kinase system to the Hsc70/DNJ-13 chaperone machinery. Speculating on the relevance, this interaction might be important when transferring a kinase from the Hsc70 chaperone system to the Hsp90 chaperone system for further maturation. While this transfer process has been well established for the maturation of the GR receptor and other steroid binding receptors with the help of the cofactor Hop/STI-1^50,51^, it is less well established for the kinase clients.

Interestingly an interaction of DNJ-13 s human orthologue DNAJB5 with Hop was identified before^50^ and the relevance of Hsc70 for the maturation of kinases has been likewise observed^52^. A transfer of the client might therefore be required to coordinate the two chaperone systems. Alternatively, the interaction might be relevant for the degradation of the kinase after release from CDC-37, where likewise the Hsc70-system could participate. In any setting, the identification of this direct interaction between the two co-factors provides insight into the mechanisms of chaperone interaction during client processing.

We set out to define the interaction sites between CDC-37 and DNJ-13. To determine the interaction sites on DNJ-13 and CDC-37 an isotope-labeled crosslink for mass spectroscopy and deletion mutants of the two proteins were investigated. One single crosslink hints to an interaction of the C-terminal domain II of DNJ-13 with the N-terminus of CDC-37 (Fig. 5). Analytical ultracentrifugation experiments with CDC-37ΔN and CDC-37ΔC mutants and full-length DNJ-13 show in contrast to the crosslink, that CDC-37ΔN binds stronger to DNJ-13 than CDC-37ΔC. This indicates that binding may occur on several sites with a stronger contribution coming from the C-terminus of CDC-37. This would contribute another complex interaction mode for CDC-37, which was found to interact with kinases and HSP-90 also via multiple interaction sites, despite the N-terminus being known as the kinase binding site^1^.

## Supplementary Information

Supplementary Figures.

## Data Availability

All data are fully available without restriction.
