# Supplementary material for: Nematode CDC-37 and DNJ-13 form complexes and can interact with HSP-90
Source: Sci Rep. 2021 Nov 1;11:21346. doi: 10.1038/s41598-021-00885-4 (PMC8560915; doi:10.1038/s41598-021-00885-4)
Supplement: Supplementary file 1 — Supplementary Figures. [file 41598_2021_885_MOESM1_ESM.docx]

**Nematode CDC-37 and DNJ-13 form complexes and can interact with HSP-90**

Lukas Schmauder^1^, Eva Absmeier^1^, Alexander Bepperling^1^, Katalin Barkovits^2,3^, Katrin Marcus^2,3^ & Klaus Richter^1,*^

^1^ Center for Integrated Protein Research at the Department of Chemistry, Technische Universität München, Lichtenbergstr. 4, 85748 Garching

^2^ Medizinisches Proteom-Center, Ruhr-Universität Bochum, Gesundheitscampus 4, 44801, Bochum, Germany.

^3^ Medical Proteome Analysis, Center for Protein Diagnostics (PRODI), Ruhr-University Bochum, Gesundheitscampus 4, 44801, Bochum, Germany.

^*^ Corresponding author. E-Mail: [klaus.richter@richterlab.de](mailto:klaus.richter@richterlab.de), Tel: +49-89-289-13342

**Supplemental Figures.**

**Supplemental Figure 1a.**

**
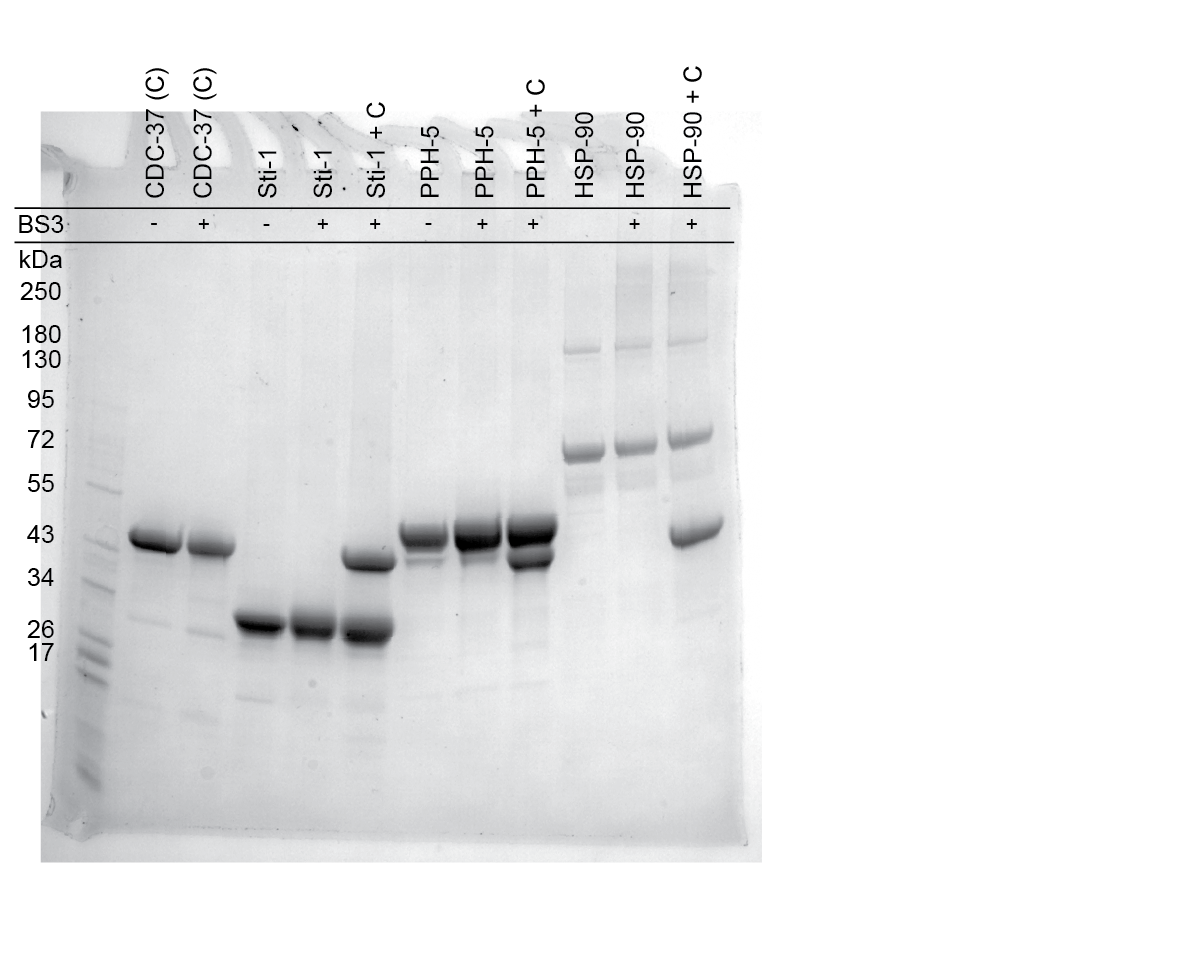
**

**Supplemental Figure 1a.:** Uncropped image of used for the creation of the left part Figure 1a. SDS-PAGE of CDC-37 (C) with and without the crosslinking reagent BS3, as indicated together with the cofactors Sti1, PPH-5, and HSP-90.

**Supplemental Figure 1b.**


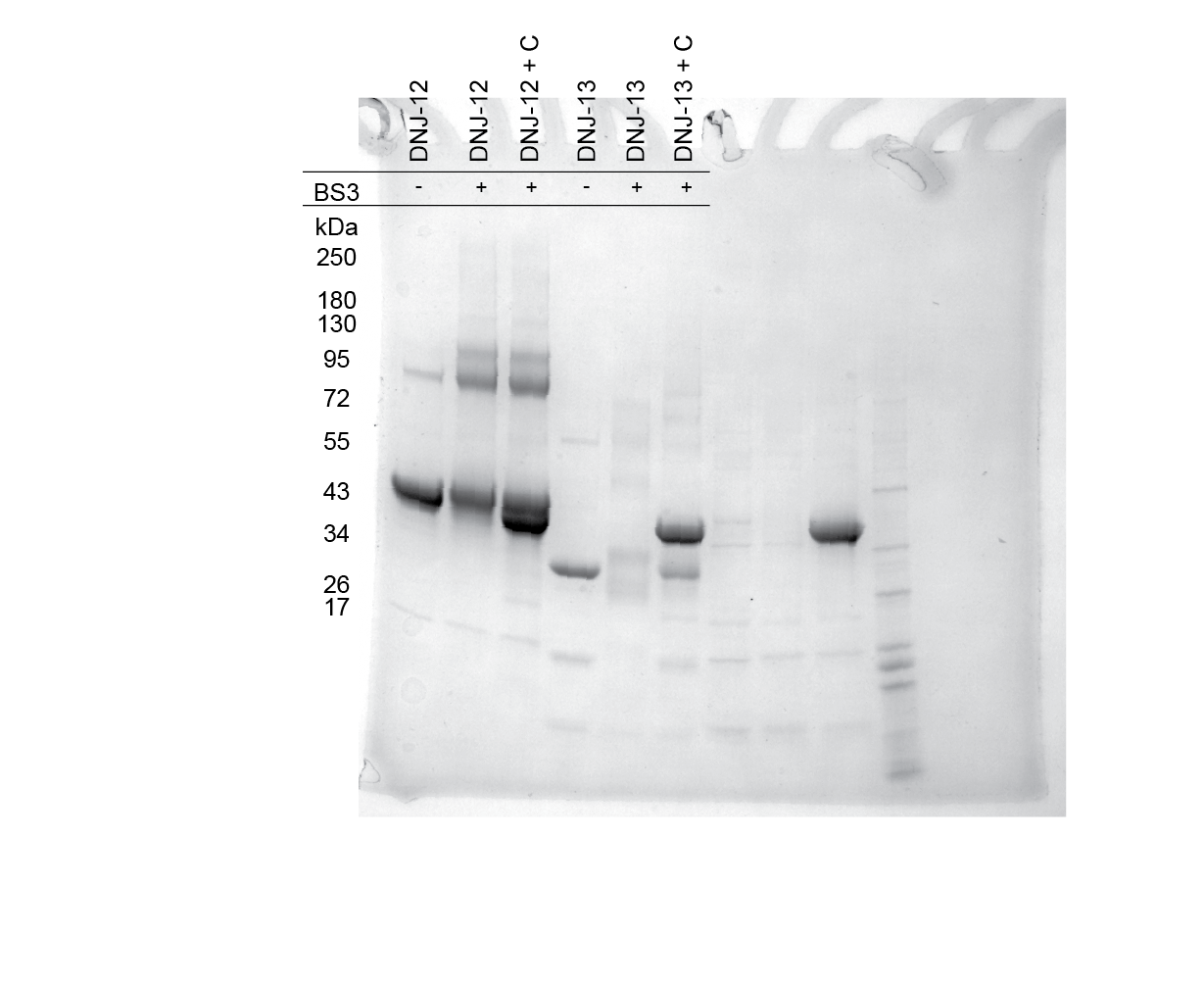


**Supplemental Figure 1b.:** Uncropped image of used for the creation of the right part of Figure 1a. SDS-PAGE of CDC-37 (C) crosslinked with and without the reagent BS3, as indicated together with the cofactors DNJ-12 and DNJ-13.

**Supplemental Figure 2.**

**
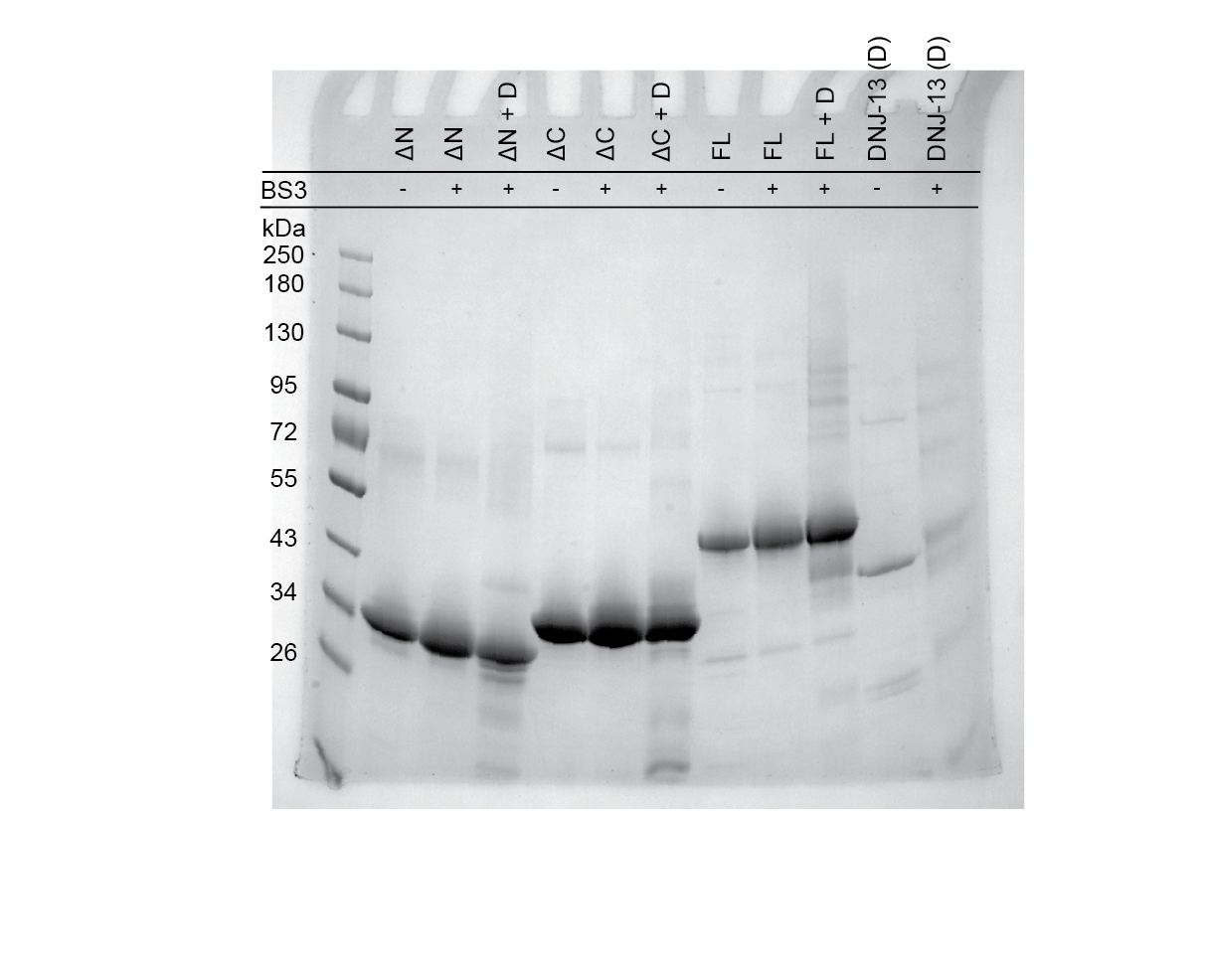
**

**Supplemental Figure 2.:** Uncropped image of used for the creation of Figure 3a. SDS-PAGE of crosslinked DNJ-13 (D) together with CDC-37ΔN (ΔN), CDC-37ΔC (ΔC) or full-length CDC-37 (FL).

**Supplemental Figure 3.**

**
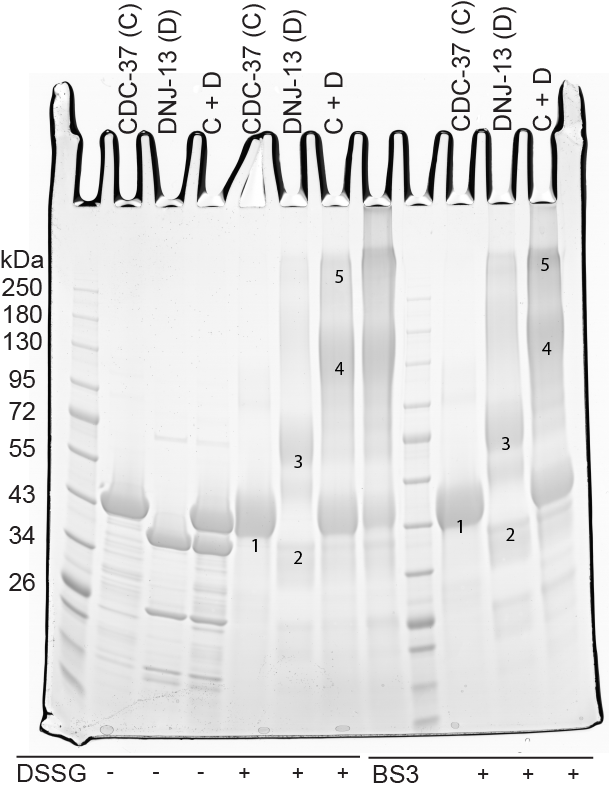
**

**Supplemental Figure 3.:** Uncropped image of used for the creation of Figure 5a. Samples used for MS-analysis and crosslinked with DSSG are shown on the left side, whereas samples crosslinked with BS3 are shown on the right. 1 = CDC-37 monomer, 2 = DNJ-13 monomer, 3 = DNJ-13 dimer, 4 = CDC37•DNJ13 complex made of one CDC-37 and two DNJ-13 molecules, 5 = complex consisting of two CDC37•DNJ13 complexes.
